# Googling Stroke ASPECTS to Determine Disability: Exploratory Analysis from VISTA-Acute Collaboration

**DOI:** 10.1371/journal.pone.0125687

**Published:** 2015-05-11

**Authors:** Richard Beare, Jian Chen, Thanh G. Phan, VISTA-Acute Collaboration

**Affiliations:** 1 Stroke Unit, Stroke and Aging Research Group, Monash University, Melbourne, Australia; 2 Monash Health and Stroke and Aging Research Group, Monash University, Melbourne, Australia; 3 Developmental Imaging, Murdoch Childrens Research Institute, Royal Childrens Hospital, Melbourne, Australia; Institute of Psychology, Chinese Academy of Sciences, CHINA

## Abstract

The summed Alberta Stroke Program Early CT Score (ASPECTS) is useful for predicting stroke outcome. The anatomical information in the CT template is rarely used for this purpose because traditional regression methods are not adept at handling collinearity (relatedness) among brain regions. While penalized logistic regression (PLR) can handle collinearity, it does not provide an intuitive understanding of the interaction among network structures in a way that eigenvector method such as PageRank can (used in Google search engine). In this exploratory analysis we applied graph theoretical analysis to explore the relationship among ASPECTS regions with respect to disability outcome. The Virtual International Stroke Trials Archive (VISTA) was searched for patients who had infarct in at least one ASPECTS region (ASPECTS ≤9, ASPECTS=10 were excluded), and disability (modified Rankin score/mRS). A directed graph was created from a cross correlation matrix (thresholded at false discovery rate of 0.01) of the ASPECTS regions and demographic variables and disability (mRS>2). We estimated the network-based importance of each ASPECTS region by comparing PageRank and node strength measures. These results were compared with those from PLR. There were 185 subjects, average age 67.5± 12.8 years (55% Males). Model 1: demographic variables having no direct connection with disability, the highest PageRank was M2 (0.225, bootstrap 95% CI 0.215-0.347). Model 2: demographic variables having direct connection with disability, the highest PageRank were M2 (0.205, bootstrap 95% CI 0.194-0.367) and M5 (0.125, bootstrap 95% CI 0.096-0.204). Both models illustrate the importance of M2 region to disability. The PageRank method reveals complex interaction among ASPECTS regions with respects to disability. This approach may help to understand the infarcted brain network involved in stroke disability.

## Introduction

**Fig 1:**
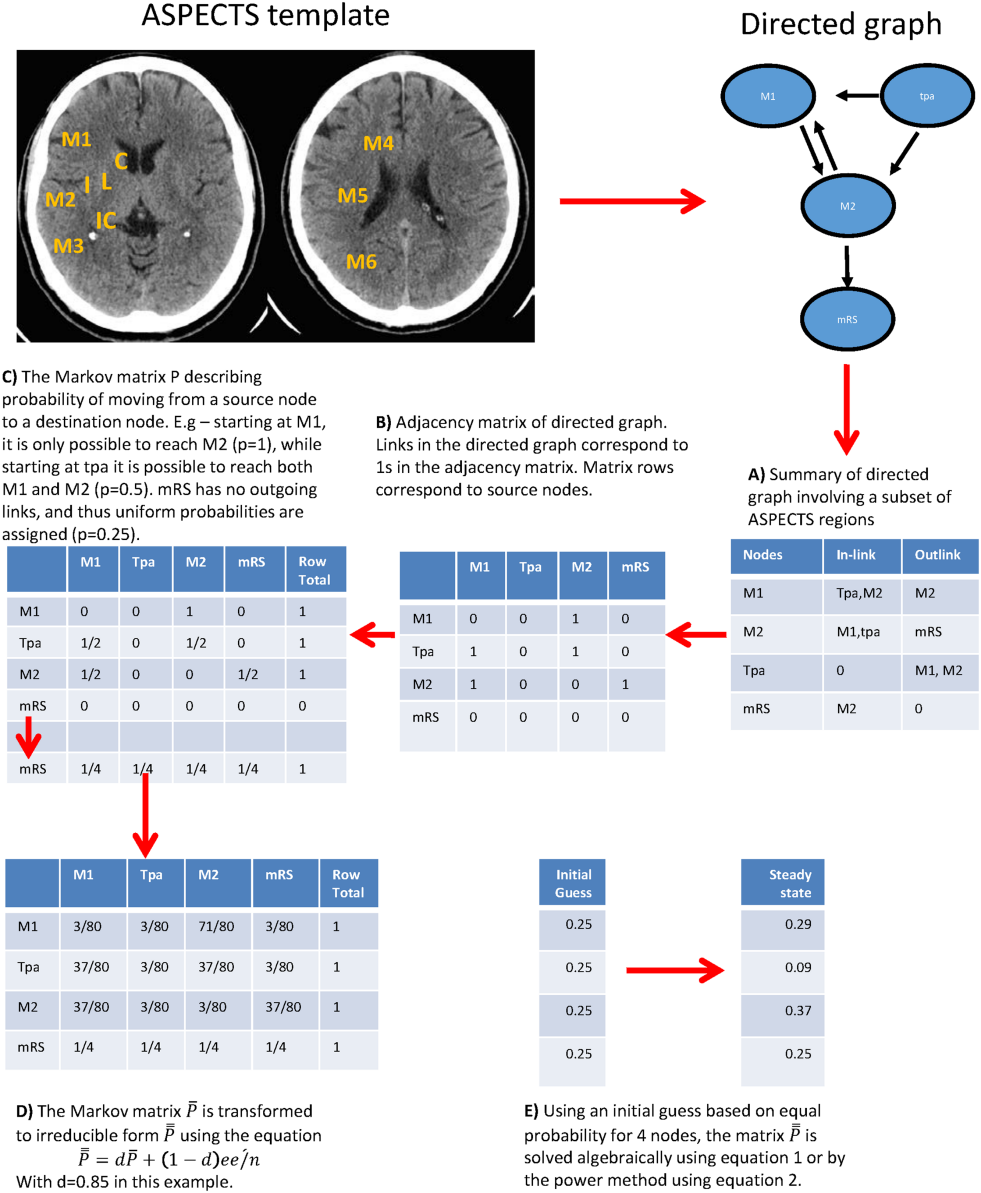
ASPECTS template and example of PageRank calculation. A working example of PageRank calculation is provided. There are 4 nodes here with their relationship provided by the arrows. The ASPECTS template is provided in the background to illustrate the location of the regions. The regions are considered as a network leading to disability. The PageRank is viewed as the frequency of being visited by a random surfer in steady state. It is a form of popularity election contest in which each node has one counted vote. M2 has the highest PageRank among ASPECTS regions. This effect was modified by the use of tpa but not by age.

Stroke remains the second leading cause of adult disability and death worldwide [1, 2]. In the acute and subacute phase, clinicians are often asked to provide long-term prognosis and potential rate of recovery for patients. Using acute CT scans, researchers have previously suggested that the Alberta Stroke Program Early CT Score (ASPECTS) system could be used to estimate functional outcome in patients receiving recombinant tissue plasminogen activator (Tpa) [3–7]. This semi-quantitative tool uses the dichotomized summed score for prognostication [3]. This type of analysis does not utilize the anatomical information available in the ASPECTS template (Fig 1). This issue has occurred because traditional regression methods requires modification to handle the issue of collinearity (relatedness) among brain regions (adjacent brain regions have related function and share vascular territory) [8].

This concept of relatedness of brain regions is consistent with current concepts on brain function. Investigators propose widely distributed network of connected brain regions contributing to observed neurological function [9–11]. Central to this idea is that neurological function is the sum total of all regions in the connected brain network rather than a single location. Conversely, persistent neurological deficit may arise from the cumulative effect of multiple lesions interrupting effective communication in this network [12]. This idea has led to several type of analyses for evaluating connectivity [10]. In a functional MRI experiment, temporal correlation of BOLD signal among different brain regions suggests that those regions form a functional connectivity type of network. Effective connectivity is suggested by the presence of structural connection (diffusion weighted imaging or anatomical dissection) among brain regions. With respect to the ASPECT regions, their membership of the middle cerebral artery territory suggest at least a form of ‘arterial’ connectivity; the term functional connectivity is not used due to the nature of the experimental condition in this case being stroke and the structural imaging used. An effective connectivity may exist between adjacent ASPECTS regions such as M2 and M3 but not necessarily between M1 and M6 (Fig 1).

There are several covariance based methods developed to explore brain connectivity. Partial least squares and partial least squares with penalized logistic regression (PLS-PLR) are methods that have been used to understand the covariance between neurological deficit and imaging abnormalities [13, 14]. However these methods are more appropriate for voxel based analysis where the columns (representing each voxel) of data are as large as 902629 voxels. Penalized logistic regression (PLR) is a related technique to PLS-PLR that was developed for analysis of interactions in genetic data [8]. It has been used for analysis of parcellated regions or regions of interest data such as ASPECTS regions [5]. It provides results in terms of β coefficient related to specific infarct locations in a manner that is intuitively understood by clinicians whereas the human observer would have difficulty integrating per-voxel β coefficients from PLS-PLR. A drawback in one version of this method is that it handles only 5 variables at given time [8], a potential issue with imaging data.

Graph based methods have emerged as tools for interpreting and analysing connected network structures and in this case network structures associated with disability. These types of analysis are attractive because they assess the connectedness of each region of interest (ROI) with respect to other ROIs over the entire brain network. Eigenvector centrality methods have been used to explore connectedness of brain regions [15]. PageRank is a variant of eigenvector centrality and is an ideal method for analysis of directed graph (the edges between adjacent nodes (regions) have direction). This method was initially developed as the basis of the search engine for Google [16]. PageRank offered a considerable improvement over pure text based methods in ranking search results, and had the advantage of being content independent (i.e. the search is based on links between the web pages). PageRank emphasises web pages based on the number of links directed to a page and the importance of the sources of those links. Thus a small number of links from influential pages can greatly enhance the importance of the destination page. In this exploratory analysis we use PageRank method to uncover the relationship of individual ASPECTS regions with respect to each other and disability outcome.

## Methods

The VISTA archives contain data from clinical trials including both ischemic and hemorrhagic stroke trials [17]. The data are released in de-identified manner so that the trials and treatment allocations are not known. We searched the *VISTA archives* for the following: imaging data: infarct, ASPECTS readings, physiological variables (systolic blood pressure, blood sugar level), demographic data (age, gender), risk factors (hypertension, diabetes, antiplatelet drugs) and 3 months disability outcome data (modified Rankin score [mRS]). The modified Rankin Score provides a measure of disability, with a score of 0 indicating no disability, 5 representing vegetative state and 6 representing death [18]. We dichotomized the modified Rankin Scale (mRS) to define functional independence function (score 0–2) and dependence or death (score 3–6).

### VISTA

We tried not to duplicate previous publications from other investigators using the VISTA data [19, 20] or planned studies by other VISTA collaborators. First we submitted a written project to the VISTA committee. To ensure that the project was not a duplication of existing VISTA projects, ours was checked against other competing projects. In this study, we compare graph theory based methods, specifically PageRank, against penalized logistic regression to determine the association between infarct location and disability.

### ASPECTS template

The ASPECTS template is described at two vertical levels: at the level of the thalamus and striatum (M1–M3, caudate, putamen, internal capsule, insular), and those superior to this level (M4–M6) [3]. One point is deducted for partial or total involvement by acute infarction in any of the 10 regions. An ASPECTS rating of 10 represents no visible infarction and a score of 0 represents diffuse ischemia throughout the MCA territory [3].

### Graphical network

A graph consists of vertices (nodes) and edges (links). The nodes in this case are variables such as ASPECTS regions, demographic and risk factors. The edges can have direction in which case it is known as directed graph (digraph). Edge direction indicates flow from a source node to a destination node. The nodes and their directed edges can be represented as an adjacency matrix. A directed graph has an asymmetric adjacency matrix. A directed graph was created from the ASPECTS regions and demographic variables and disability (mRS>2). In this analysis, there is no *a priori* assumption about the relationship among the ASPECTS regions from a point of view of fibre connection and each region is assumed to connect to each other. The graph model was constructed as follows:

- Common features of Models 1 and 2
  - ASPECTS regions have bidirectional relationships with one another.
  - ASPECTS regions have a bidirectional relationship with disability.
  - Connections between ASPECTS regions are weighted by distance matrix, based on calculation of city block distance. In this case, M1 and M2 are described by unit length of one. M1 and M3 are separated by unit length of two. M4 and M3 are separated by unit length of three (M4 to M1 then onto M2 and finally to M3).
  - Demographics and risk factors do not have relationships with one another.
- Unique features
  - Model 1-demographic and risk factor variables have a bidirectional relationship with ASPECTS regions but do not have a bidirectional relationship with disability.
  - Model 2- demographic and risk factor variables have a bidirectional relationship with disability.
- The above models were re-evaluated using an adjacency weighting scheme instead of the city block distance weighting scheme. In this case, M1 and M2 and M1 and M5 are separated by unit length of one. M3 and M4 or M1 and M6 are not adjacent. This analysis was performed to assess the impact of weighting scheme on the results.

All variables used in this study are binary. Edge strength was computed using cross correlation scores between nodes/variables. Pearson correlation score between binary variables is equivalent to the Phi coefficient, or mean square contingency coefficient, which are related to the chi-square statistic. Edges were removed from the correlation-based graph (i.e. the cross correlation matrix was thresholded) if the p-value for the correlation did not survive false discovery rate (FDR) correction for multiple comparisons (p<0.01).

### Centrality measures

Centrality measures assign a measure of “importance” to nodes and can therefore indicate whether some nodes are more critical than others in a given network. When network nodes represent variables, centrality measures may indicate relevance of variables to a model.

The simplest centrality measure, degree centrality, is the count of links for each node and is a purely local measure of importance. Node strength, used in weighted networks, is the sum of weights of edges entering or leaving (or both) the node. Other measures, such as betweenness centrality, describe more global structure—the degree of participation of a node as conduit of information between other nodes in a network.

PageRank is one member of a family of graph eigenvector centrality measures, all of which incorporate the idea that the score of a node depends, at least in part, on the scores of neighbors connecting to the node. Thus a page may have a high PageRank score if many pages link to it, or if a few important or authoritative pages link to it. Others include eigenvector centrality (which works best with undirected graphs), alpha centrality and Katz centrality. PageRank uses a different scaling for connections (by the number of links leaving the node) and importance is based on incoming connections rather than outgoing connections. Eigenvector centrality measure a node’s centrality in terms of node parameters and centrality of neighboring nodes [21]. PageRank has several differences with respect to other eigenvector centrality methods, expanded below, which make it better suited for digraphs. PageRank was originally described in terms of a web user/surfer randomly clicking links, and the PageRank of a web page corresponds to the probability of the random surfer arriving at the page of interest [16]. The model of the random surfer used in the PageRank computation includes a damping factor, which represents the chance of the random surfer becoming bored and selecting a completely different page at random (teleporting to a random page). Similarly, if a page is a sink (i.e. has no outgoing links), then the random web surfer may click on to a random page.

A number of different approaches are available for computing the PageRank for nodes in a network [22]. The conceptually simplest is to assign an equal initial score to each node, and then iteratively update PageRank scores using the following formula:

$$G\left(n_{i},t_{0}\right)\;=\;k$$

$$G\left(n_{i},t_{l+1}\right)\;=\;\frac{1-d}{G}+d\sum {}_{n_{j}\in N\left(n_{i}\right)}\frac{G\left(n_{j},t_{l}\right)}{D\left(n_{j}\right)}$$ (1)

where n_1_ to n_G_ are the nodes, d is the damping factor, k is the constant used to initialize the PageRank score, D(n_j_) is the number of links leaving node j and G is the PageRank.

Alternatively, the connectivity matrix representing the graph may be converted to a stochastic and irreducible matrix in two steps [22]:

- providing sink nodes (no outgoing links) with a uniform distribution of outgoing links. This corresponds to the “teleporting” step above. Each sink node is linked to all other nodes with equal probability. After this step the matrix is stochastic.
- Combining with a stochastic perturbation matrix. This step incorporates the damping factor:
  $$\bar{\bar{P}}=d\bar{P}+(1-d)\frac{ee^{T}}{n}$$
  where $\overset{-}{P}$ is the stochastic matrix incorporating “teleporting”, n is the number of nodes, d is the damping factor and e is a column matrix of ones.

The PageRank is then given by the solution to:

$$G\bar{\bar{P}}=G$$ (2)

Thus the PageRank scores correspond to the values of the dominant Eigenvector of the modified adjacency matrix. The dominant Eigenvector can be computed efficiently using power iteration methods. Finally, the problem may be cast as linear system, which has numerical advantages. The tool used for this article, igraph/prpack, (R statistical Foundation) employs the linear system approach.

A modification of PageRank, known as *Relative* PageRank, was introduced by [23]. Relative PageRank computes the importance of nodes relative to a set of *root* nodes. This is achieved by introducing a *personalization vector* that serves to make the root nodes the only destination for the teleporting phase. Root nodes must therefore have outgoing edges—dangling root nodes are therefore allowed to teleport to any node. We use disability outcome as the root node.

Bootstrapping was used to determine the confidence interval of the PageRank calculation.

The PageRank algorithm uses an empirically determined damping factor of 0.85. This factor may be appropriate for the World Wide Web PageRank calculation but its calculation for structural imaging data is unknown. We performed a sensitivity analysis on the damping factor to determine the optimal damping factor for structural imaging data.

### Node Strength

The strength of a node is a local measure corresponding to the sum of edge weights arriving and/or departing from that node. The equivalent measure in a binary graph is the node degree, corresponding to the count of nodes arriving and/or departing from the node. We used the sum of edge weights arriving at the node. In this form the node strength approximately corresponds to the local component of the PageRank measure. Bootstrapping, with 5000 iterations and the adjusted bootstrap percentile method, was used to determine the confidence interval of the node strength measures. Node strength measures are independent of choice of PageRank or relative PageRank.

### Combining PageRank and Node Strength

PageRank and node strength were computed for nodes connected to the outcome node. The rank of each node within the network was computed for both PageRank and node strength.

A difference in ranking between the two measures was then computed to estimate the contribution of global network structure to the PageRank score for each node:

$$W(i)=G_{r}(i)-N_{r}(i)$$

where G_r_(i) is the rank of the PageRank score for node i, N_r_(i) is the rank of the node strength for node i and *W* is the global network contribution (GNC). *W* thus provides an indication of how many positions a node may shift as a result of network structure—e.g. does a node shift from third most important to most important. A positive GNC indicates that the node’s PageRank is above the level explained by local connections, and thus possesses an importance derived from network structure.

### PLR

PLR is a form of logistic regression with a bias/penalty factor added to prevent overfitting of the model [8]. There are different strategies used for choosing the bias factor such as linear estimator *L1* (unconstrained minimization of the least-squares penalty), curved estimator *L2* (quadratic penalization of the parameter estimate of the maximum likelihood) and combined *L1* and *L2* penalization. The PLR method used here performs L2 penalization. This program assesses interaction by applying the asymmetric hierarchy principle [24]. Any predictor retained in the model can form interactions with others that are already in the model and those that are not yet in the model. The choice of predictors to be added/deleted to the stepwise regression was based on the cost complexity statistic. We used a forward and backward stepwise PLR that used all the ASPECTS regions in the analysis, calling on the penalized function in R programming environment. In this analysis, we have specified a maximum of five terms to be added to the selection procedure.

## Results

In this sample, we only included subjects with abnormal ASPECTS score i.e ASPECTS ≤9. As such subjects with ASPECTS = 10 were excluded from the analysis for reasons provided above. There were 185 subjects, average age 67.5± 12.8 years (55.1% males). In this sample, 78% of the subjects had received Alteplase. Hypertension and diabetes were present in 70.3% and 16.2% respectively. Poor Rankin disability score were present in 67.6% of subjects. Frequency of involvement of ASPECTS regions and univariate regression between regional involvement and outcome and demographic factors and outcome are summarised in Table 1.

**Table 1:** Univariable regression of poor outcome (modified Rankin Score >2 against ASPECTS regions.

	*Frequency of ischemic changes (%)*	*Odds ratio and 95% CI*	*P value*
Caudate	17.8	1.27 (0.51–2.65)	0.773
Internal capsule	43.2	1.88 (0.83–4.68)	0.149
Insula	60.5	1.56 (0.83–2.91)	0.166
Lentiform	43.2	1.22 (0.65–2.29)	0.538
m1	23.2	3.06 (1.34–7.95)	0.012
m2	26.5	5.98 (2.42–18.09)	0.000
m3	15.7	3.50 (1.28–12.33)	0.026
m4	9.20	0.87 (0.31–2.63)	0.792
m5	30.8	1.51 (0.77–3.10)	0.237
m6	13.0	6.19 (1.74–39.53)	0.016
Age (mean)	67.5	1.09 (1.06–1.12)	0.000
Gender (Male)	55.1	0.49 (0.25–0.93)	0.030
Tpa	78.4	0.13 (0.03–2.72)	0.000
Diabetes	16.2	2.75 (1.07–8.51)	0.051
Hypertension	70.3	3.18 (1.64–6.21)	0.001

PageRank analysis: demographic variables and risk factor variables have no direct relationship with disability-city block weighting scheme.

**Fig 2:**
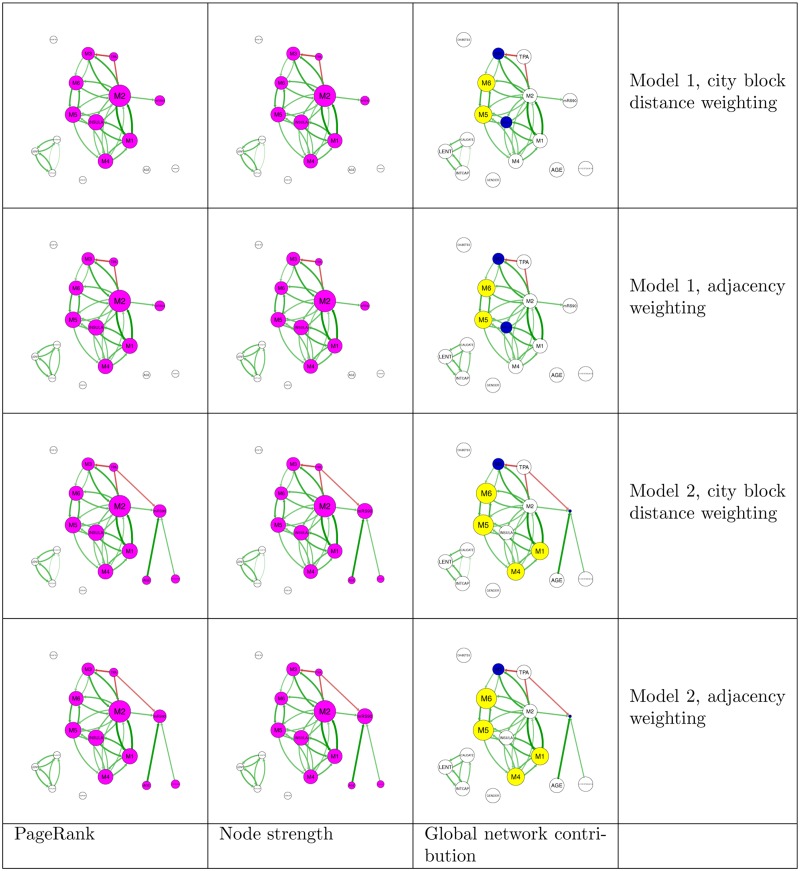
PageRank, node strength and global network contribution for all combinations of model and weighting. Pink nodes indicate the connected component including outcome (mRS90). Green edges indicate positive correlations and red edges indicate negative correlations. Higher absolute correlation is indicated by brighter and thicker edges. Yellow nodes have greater PageRank ranking than strength ranking. Blue nodes have lower PageRank ranking than strength ranking. Weighting schemes lead to minimal changes in node measures.

In this model 1 (demographic variables having no direct connection with disability and FDR <0.01), the highest PageRank node was M2 (0.225, 95% CI = 0.215 to 0.347), second was M5(0.135, 95% CI = 0.106 to 0.234), closely followed by insula (0.131, 95% CI = 0.104 to 0.251) and M1 (0.125, 95% CI = 0.097 to 0.283). The node with the highest strength was M2 (0.250, 95% CI = 0.232 to 0.409), second were insula (0.133, 95% CI = 0.102 to 0.234) and M5 (0.133 95% CI = 0.089 to 0.197). The nodes with the highest GNC were M5 and M6, both with PageRank scores 1 place above strength (95% CI = -1.0 to 3.0 and -2.0 to 3.0) place as a result of PageRank. M3 and insula decreased ranking by one place (95% CI = -5.0 to 1.0 and -4.0 to -1.0) as a result of PageRank. Other nodes did not change ranking (Fig 2).

### Relative PageRank analysis: demographic variables and risk factor variables have no direct relationship with disability-city block weighting scheme

In this model 1 (demographic variables having no direct connection with disability and FDR <0.01), the highest relative PageRank node was M2 (0.236, 95% CI = 0.219 to 0.385), second was M5 (0.141, 95% CI = 0.110 to 0.245) followed by insula (0.138, 95% CI = 0.106 to 0.267) and M1 (0.131, 95% CI = 0.100 to 0.295). The nodes with the highest GNC were M5 and M6, all having relative PageRank scores 1 place above strength scores (95% CI = -1 to 4 and -1 to 5.0) and TPA increasing by 0.5 places (95% CI = -1.0 to 0.5). M3 and insula decreased ranking by 1 place (95% CI = -5.0 to 0.0 and -4.0 to -1.0)

### PageRank analysis: demographic variables and risk factor variables have no direct relationship with disability-adjacency weighting only

In this model 1 (demographic variables having no direct connection with disability and FDR <0.01), the highest PageRank node was M2 (0.225, 95% CI = 0.216 to 0.369), second was M5 (0.135, 95% CI = 0.103 to 0.252) followed by insula (0.131, 95% CI = 0.105 to 0.229) and M1 (0.125, 95% CI = 0.093 to 0.370). The node with the highest strength was M2 (0.250, 95% CI = 0.229 to 0.424), second were insula (0.133, 95% CI = 0.103 to 0.225) and M5 (0.133, 95% CI = 0.084 to 0.202) followed by M1 (0.128, 95% CI = 0.086 to 0.210). The nodes with the highest GNC were M5 and M6, with PageRank scores 1 place above strength scores (95% CI = -1.0 to 3.0 and -1.0 to 3.0). M3 and insula decreased ranking by 1 place (95% CI = -5.0 to 1.0 and -4.0 to -1.0). Other nodes did not change ranking.

### Relative PageRank analysis: demographic variables and risk factor variables have no direct relationship with disability-adjacency weighting only

In this model 1 (demographic variables having no direct connection with disability and FDR <0.01), the highest relative PageRank node was M2 (0.236, 95% CI = 0.220 to 0.407), second was M5 (0.141, 95% CI = 0.107 to 0.263), followed by insula (0.138, 95% CI = 0.108 to 0.249) and M1 (0.131, 95% CI = 0.096 to 0.411). The nodes with the highest GNC were M5 and M6, with PageRank scores 1 place above strength scores (95% CI = -1.0 to 5.0) and TPA with an increase of 0.5 (95% CI = -1.0 to 0.5). M3 and insula decreased ranking by 1 place using relative PageRank (95% CI = -5.0 to 0.0, -4.0 to -1.0).

### PageRank analysis: demographic variables and risk factor variables have a unidirectional relationship with disability-city block weighting scheme

**Fig 3:**
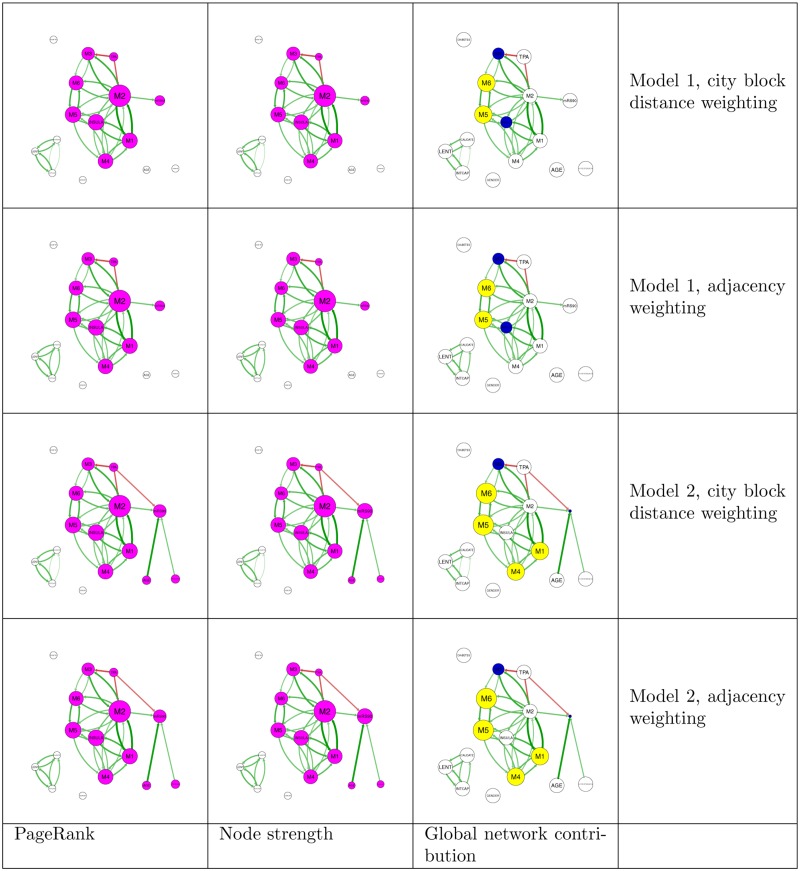
Relative PageRank, node strength and global network contribution for all combinations of model and weighting. Pink nodes indicate the connected component including outcome (mRS90). Green edges indicate positive correlations and red edges indicate negative correlations. Higher absolute correlation is indicated by brighter and thicker edges. Yellow nodes have greater PageRank ranking than strength ranking. Blue nodes have lower PageRank ranking than strength ranking. Weighting schemes lead to minimal changes in node measures.

In this model 2 (demographic variables having direct connection with disability and FDR <0.01), the highest PageRank node was M2 (0.205, 95% CI = 0.194 to 0.367), second was M5 (0.125, 95% CI = 0.096 to 0.204) followed by insula (0.122, 95% CI = 0.088 to 0.200) and M1 (0.116, 95% CI = 0.090 to 0.225). The node with the highest strength was M2 (0.225, 95% CI = 0.210 to 0.377), second was mRS90 (0.126, 95% CI = 0.073 to 1.00), followed by insula (0.120, 95% CI = 0.091 to 0.188) and M5 (0.120, 95% CI = 0.075 to 0.174). The nodes with the highest GNC were M5 and M6, with PageRank scores 2 places above strength scores (95% CI = 0.0 to 8.0 and 1 to 8). mRS90 decreased ranking by 5 places (95% CI = -14.7 to -3.0) and M3 by 1 place (95% CI = -4.0 to 1.0). Other nodes did not change ranking (Fig 3).

### Relative PageRank analysis: demographic variables and risk factor variables have a unidirectional relationship with disability-city block weighting scheme

In this model 2 (demographic variables having direct connection with disability and FDR <0.01), the highest relative PageRank node was M2 (0.224, 95% CI = 0.21003926 to 0.450), second was M5 (0.136, 95% CI = 0.105 to 0.227), followed by insula (0.133, 95% CI = 0.098 to 0.220) and M1 (0.126, 95% CI = 0.098 to 0.274). The nodes with the highest GNC were M5 and M6, with PageRank scores 1 place above strength scores (95% CI = -1.0 to 4.8, -1.0 to 7.0) and TPA and hypertension, with PageRank scores 0.5 places above strength scores (95% CI = -1.5 to 0.5, -0.5 to 0.5).

### PageRank analysis: demographic variables and risk factor variables have a unidirectional relationship with disability-adjacency weighting scheme

In this model 2 (demographic variables having direct connection with disability and FDR <0.01), the highest PageRank nodes were M2 (0.205, 95% CI = 0.194 to 0.321), second was M5 (0.125, 95% CI = 0.095 to 0.202) followed by insula (0.122, 95% CI = 0.092 to 0.212) and M1 (0.116, 95% CI = 0.089 to 0.289). The node with the highest network strength was M2 (0.225, 95% CI = 0.211 to 0.356) second was mRS90 (0.126, 95% CI = 0.051 to 0.649) followed by insula (0.120, 95% CI = 0.092 to 0.198) and M5 (0.120, 95% CI = 0.073 to 0.173). The nodes with highest GNC were M5 and M6, both having PageRank scores 2 places above strength scores (95% CI = 0.0 to 6.0 and 1.0 to 5.0). mRS90 decreased rank by 5 places (95% CI = -9.0 to -3.0).

### Relative PageRank analysis: demographic variables and risk factor variables have a unidirectional relationship with disability-adjacency weighting scheme

In this model 2 (demographic variables having direct connection with disability and FDR <0.01), the highest PageRank nodes were M2 (0.224, 95% CI = 0.213 to 0.345) second was M5 (0.136, 95% CI = 0.103 to 0.231), followed by insula (0.133, 95% CI = 0.102 to 0.238) and M1 (0.126, 95% CI = 0.095 to 0.322). Nodes with highest GNC were M5 and M6, with relative PageRank scores two places above strength scores (95%CI = -1.0 to 4.0) and M1 and M4 with PageRank scores 1 place above strength scores (95% CI = -3.0 to 1.0, -4.5 to 0.0).

### PageRank—Sensitivity analysis by varying damping factor

**Fig 4:**
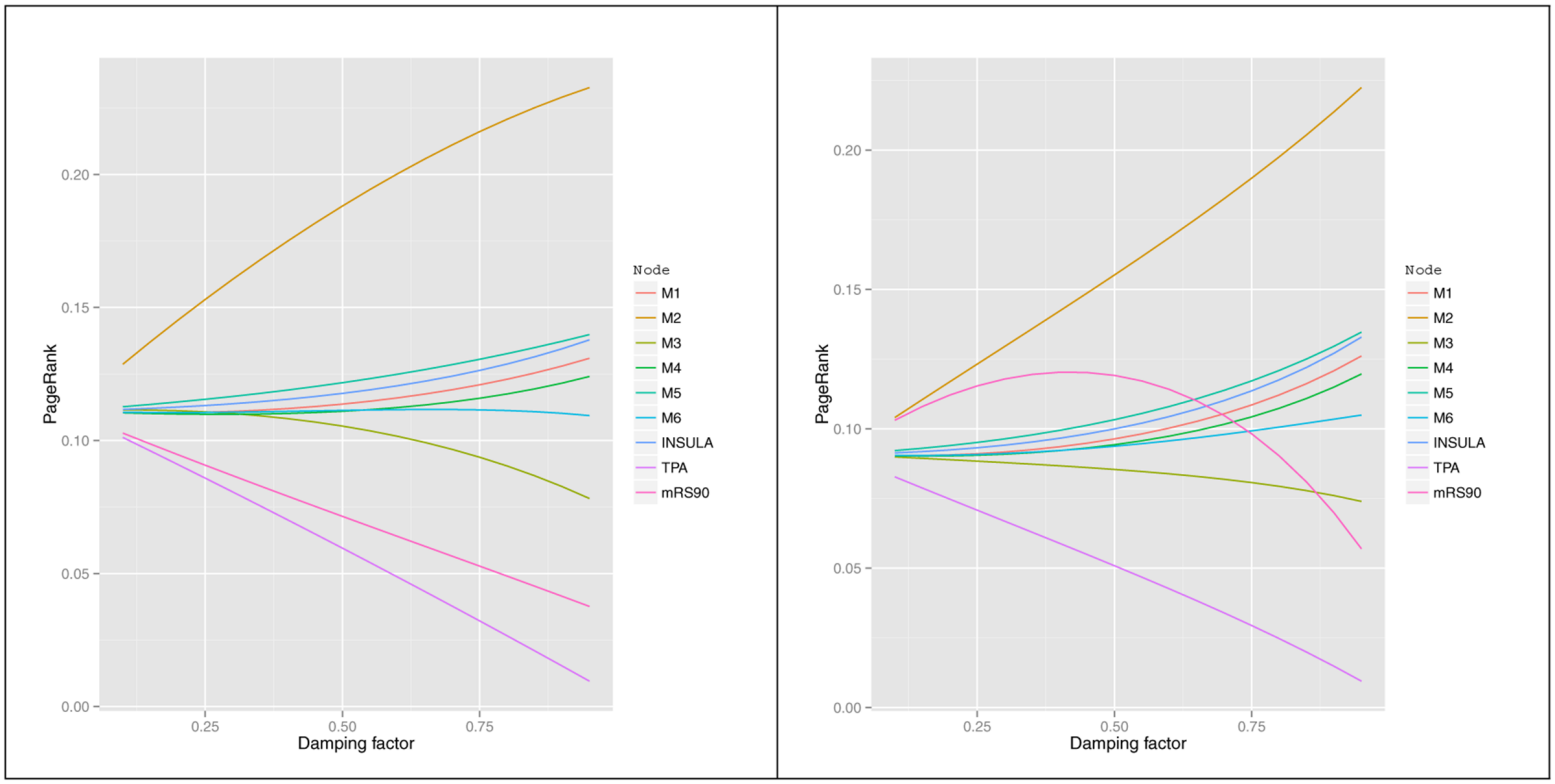
Change in PageRank score with damping factor for model 1 (left) and model 2 (right) with distance weighting.

The sensitivity analysis for model 1 (demographic variables having direct connection with disability and FDR <0.01) showed that the PageRank of the ASPECTS regions separated from each other after the damping factor was increased above 0.75. At this damping factor, the demographic variables rose dramatically (see Fig 4). This was also the case for model 2 (demographic variables having direct connection with disability and FDR <0.01). In the case of relative PageRank, the node mRS follows a curve plot because the other node are calculated relative to it.

### PLR analysis

Variables that were significantly associated with poor disability outcome included: M2 (beta coefficient 1.285, 95%CI 0.214–2.357, p = 0.02), age (beta coefficient 0.078, 95% CI 0.0468–0.1101, p = 0.01) and tpa (beta coefficient -1.713, 95% CI -3.051 -0.374, p = 0.01).

## Discussion

We have taken advantage of the anatomical mapping strategy employed in the ASPECTS template to evaluate a different method for exploring the association between brain regions and outcome. This was achieved by using the method of PageRank, a variant of eigenvector centrality based method that has been successfully used by Google in its search engine [16, 22]. This method provides similar results to PLR that we had previously explored for prediction of stroke outcome [5]. Our finding is intended to be used for exploring models for understanding the relationship between infarct location and outcome. The methodology employed here is not restricted for CT scans but can also be evaluated with other imaging modalities such as CT angiography source images, CT perfusion images or diffusion-weighted imaging, diffusion tensor imaging [25].

### Eigenvector method and regression method

In this study, the eigenvector centrality based method was used to develop a model for understanding stroke disability. It was not intended as a regression model and in this respect, regression based methods such as PLR have an advantage over this method by providing regression coefficients, such as the results provided here. Rather, the graphical approach permits display of the influential nodes (ASPECTS regions) over the entire network. By contrast, the PLR method only denotes the influence of M2 region on outcome but not its influence on the other ASPECTS regions. Several regions (M1, M4, M5 and M6) have higher importance due to their position in the network over than can be attributed to direct connections. This is an example of how the PageRank approach can be used in an exploratory fashion. The choice of relative PageRank or standard PageRank did not change the nodes that were detected as having increased importance due to position within the network. The difference between PageRank and relative PageRank may increase with larger networks.

The method that we describe here is adaptable as we have shown through the exploration of different models of interactions among the variables. Such flexibility (emphasis of different directions in the relationship among variables) is not easily available with traditional regression method. A drawback is the absence of formal method for testing statistical significance with this approach. This aspect has led to our introduction of false discovery rate, as a way for removing less significant variables, when setting up the correlation matrix. Next the width of the confidence interval of the PageRank, determined by bootstrapping, was used as another way to check the PageRank estimate.

### Comparisons to other covariance based methods

Eigenvector based methods have the advantage over traditional regression method in that it can avoid the issue of collinearity and handle larger dataset [26]. This type of analysis has some similarity to other covariance based methods such as principal component regression (PCA), independent component analysis (ICA) and partial least squares (PLS) in that it is data driven. However, those methods determine node importance within the context of that particular component i.e. within the first principal component or within the second component and so on. This process is different from PageRank analysis which assesses node importance over the entire network from the principal eigenvector.

These covariance based methods involve regressing the voxels in the images and the outcome of interest. One of the disadvantages with both principal component regression [27] and partial least squares [13, 14, 28, 29] that we identified is the inability of these methods to take into account other covariates. This effect is likely to be due to the use of voxel type analysis compared to multiple regions of interests approach (eg. 10 ASPECTS regions). The presentation of an image of beta coefficients at every voxel does not lend itself easily to an understanding of the individual interactions.

### PageRank and damping factor

Investigators have assessed PageRank centrality measure against other measures such as degree centrality, subgraph centrality, eigenvector centrality and have found this measure (PageRank) to perform better than the others at capturing both global and local centrality [15]. This efficiency has been attributed to the use of the damping factor in the PageRank algorithm [15]. In this analysis, we have assessed the impact of changing the damping factor on our analysis because of the use of dangling nodes in this study. Our analysis suggests a generally smooth variation in PageRank with damping factor, with differences between nodes increasing with damping factor. Thus our choice of the empirical damping factor of 0.85 is reasonable [16]. Other investigators have made similar finding with PageRank that as damping factor approach 1, there is concentration of the PageRank in the dangling nodes [22]. One potential issue with very high damping factor with world wide web calculation of PageRank is that it is computationally expensive.

### Comparison to other ‘connectome’ analyses

In healthy subjects, ‘connectome’ analyses of brain network have assessed ‘small-worldness’, hub score, a centrality measure of the outgoing link of the node and other local centrality measures such as degree and betweenness centrality. Others have used eigenvector centrality or PageRank maps of different experimental conditions as contrast maps and compared differences at each node between two groups [15, 30]. The ‘connectome’ analysis of stroke patients represents a different challenge due to the different location of stroke and resultant neurological deficit. Investigators have circumvented this issue by performing group analysis between normal control group and a post-stroke group at each node to assess the impact of stroke on brain re-organisation [26]. The approach used in this study is different from other types of connectome analyses in that the nodes represent infarct location rather functional MRI or structural analyses of white matter tract. Our analytical approach resembles the graphical output of structural equation modelling (SEM) which uses path diagrams. Compared to other ‘soft’ modelling covariance based method such as PLS described earlier, SEM is used for causal modelling of relationship. This method is based on the linear relationship between the variance and covariance among the predictors and the dependent variables. Consequently, SEM is not originally designed to handle binary data (good and poor Rankin outcome) until recent modification [31]. The SEM analysis has limited generalisation of the result over the entire data whereas the PageRank method acts as a form of exploratory analysis and provides an overview of the relationship among the variables. For a dataset such as the one provided here, it is possible, but time consuming, to systematically perform individual analysis and set up a structural equation modelling experiment. However, with much larger dataset involving many more variables, this places enormous demand on the human experimenter to keep track of relationship among the variables.

Keeping with the theme of proof of concept, we have emphasized the method but not commented on importance of regions other than the similarity between the finding of PageRank and PLR. It is likely that a hybrid approach combining these two methods can provide user who prefer regression coefficients and others who would favour a graphical display of the relationship. This type of approach can be used to explore other types of data such as effect of diabetes on cognition and falls. It is anticipated that in the future this method may be applied to more sensitive techniques for detecting ischemia (such as diffusion-weighted/ diffusion tensor imaging, perfusion CT scans, or source images of CT angiograms) in order to provide better understanding of infarct location on disability.

### Disclosure

Prof Phan reports receiving honoraria as speakers for Genzyme, Boehringer Ingelheim and Bayer. He is on the advisory board for Genzyme on Fabry Disease.
